# Acute High Dietary Phosphorus Following Low‐Phosphorus Diet Acclimation Does Not Enhance Intestinal Fractional Phosphorus Absorption in Nephrectomized Male Rats

**DOI:** 10.1002/jbm4.10698

**Published:** 2022-11-16

**Authors:** Kendal M Burstad, Dennis P Cladis, Colby J Vorland, Meryl E Wastney, Annabel Biruete, James M Dominguez, Kalisha D O'Neill, Neal X Chen, Sharon M Moe, Kathleen M Hill Gallant

**Affiliations:** ^1^ Department of Food Science and Nutrition University of Minnesota Saint Paul MN USA; ^2^ Department of Nutrition Science Purdue University West Lafayette IN USA; ^3^ Department of Applied Health Science Indiana University School of Public Health‐Bloomington Bloomington IN USA; ^4^ Department of Medicine‐Division of Nephrology Indiana University School of Medicine Indianapolis IN USA; ^5^ Department of Nutrition and Dietetics Indiana University‐Purdue University Indianapolis Indianapolis IN USA; ^6^ Department of Anatomy and Cell Biology Indiana University School of Medicine Indianapolis IN USA; ^7^ Department of Medicine Roudebush Veterans Affairs Medicine Center Indianapolis IN USA

**Keywords:** ANIMAL MODELS, DISORDERS OF CALCIUM/PHOSPHATE METABOLISM, NUTRITION, PRECLINICAL STUDIES, PTH/VitD/FGF23

## Abstract

Dietary phosphorus restriction and phosphorus binders are commonly prescribed for patients with chronic kidney disease (CKD). However, occurrences of non‐adherence to these interventions are common. As low‐phosphorus (LP) diets have been consistently experimentally shown in vitro to increase intestinal phosphorus absorption efficiency, a bout of non‐adherence to diet or binders may cause an unintended consequence of enhanced intestinal phosphorus absorption. Thus, we aimed to determine the effect of a single bout of high‐phosphorus (HP) intake after acclimation to a LP diet. Male Sprague Dawley rats with 5/6 nephrectomy (*n* = 36) or sham operation (*n* = 36) were block‐randomized to 1 of 3 diets: LP (0.1% P w/w), HP (1.2%), or LP followed by acute HP (LPHP 0.1% then 1.2%). Phosphorus absorption tests were conducted using ^33^P radioisotope administrated by oral gavage or intravenously (iv). Although the overall two‐way ANCOVA model for intestinal fractional phosphorus absorption was non‐significant, exploratory comparisons showed intestinal fractional phosphorus absorption efficiency tended to be higher in rats in the LP compared with HP or LPHP groups. Rats in the HP or LPHP groups had higher plasma phosphorus compared with rats in the LP group, but the LPHP group was not different from the HP group. Gene expression of the major intestinal phosphate transporter, NaPi‐2b, was lower in the jejunum of rats in the LPHP group compared with rats in the HP group but not different in the duodenum. These results demonstrate that an acute HP load after acclimation to a LP diet does not lead to enhanced intestinal fractional phosphorus absorption efficiency in 5/6 nephrectomized male rats. These data provide evidence against the notion that dietary phosphorus restriction or binder use adversely increases absorption efficiency after a single instance of dietary or binder non‐adherence. However, other adverse consequences of fluctuating dietary phosphorus intake cannot be ruled out. © 2022 The Authors. *JBMR Plus* published by Wiley Periodicals LLC on behalf of American Society for Bone and Mineral Research.

## Introduction

Individuals with chronic kidney disease (CKD) develop disturbances in mineral metabolism as the disease progresses.^(^ ^1^, ^2^, ^3^, ^4^ ^)^ These disturbances lead to CKD‐mineral bone disorder (CKD‐MBD), including increased vascular calcification,^(^ ^5^, ^6^ ^)^ bone fragility fractures,^(^ ^7^, ^8^ ^)^ and mortality.^(^ ^9^, ^10^ ^)^ Abnormal phosphorus metabolism drives the development of CKD‐MBD. In the absence of pathology, phosphorus homeostasis is maintained through the actions of the intestine, bone, parathyroid gland, and kidney.^(^ ^11^ ^)^ Healthy kidneys are able to fully compensate for increased phosphorus loads by increasing urinary phosphorus excretion.^(^ ^12^ ^)^ However, as kidney function declines, urinary phosphorus excretion becomes compromised, contributing to abnormal phosphorus handling leading to elevated plasma fibroblast growth factor‐23 (FGF‐23), elevated intact parathyroid hormone (iPTH), lower 1,25‐dihydroxyvitamin D (1,25D), and eventual elevated plasma phosphorus.^(^ ^2^ ^)^ Intestinal absorption of dietary phosphorus is a major component of overall phosphorus balance. Therefore, common approaches used to manage hyperparathyroidism and hyperphosphatemia include dietary phosphorus restriction and the use of phosphate binder medications.^(^ ^11^ ^)^ However, adhering to a low‐phosphorus diet is challenging as phosphorus is widespread in the food supply, including naturally occurring sources in protein and grain foods and in food products with phosphate‐containing additives.^(^ ^13^ ^)^ Further, measurement and disclosure of phosphorus content in foods is not required on the Nutrition Facts Label, making it difficult for patients with CKD to make informed food selections and for health care practitioners to make accurate recommendations.^(^ ^14^ ^)^ Thus, bouts of non‐adherence to low‐phosphorus diets are common. In fact, in an integrative review aimed at determining dietary adherence in late‐stage CKD, Lambert and colleagues^(^ ^15^ ^)^ reported that adherence to a low‐phosphorus diet ranged from 43.5% to 84.5% across 15 studies.

There is sparse literature investigating the physiological effects of bouts of dietary phosphorus restriction non‐adherence on intestinal phosphorus absorption. However, low‐phosphorus diets have been shown to increase expression of the intestinal sodium‐phosphate co‐transporter (NaPi‐2b), which is associated with in vitro measures of greater brush border membrane vesicle phosphorus uptake^(^ ^16^, ^17^, ^18^, ^19^, ^20^ ^)^ or increased phosphate flux.^(^ ^21^ ^)^ This suggests that dietary phosphorus restriction or binder use may have an adverse consequence by enhancing intestinal phosphorus absorption if there is one or multiple bouts of non‐adherence with diet or binders. A study by Giral and colleagues^(^ ^16^ ^)^ measured in vitro intestinal phosphorus uptake efficiency in isolated brush border membrane vesicles (BBMV) from healthy rats fed either a low‐phosphorus diet for the duration of the study or acutely switched to a high‐phosphorus diet on the last day. Unexpectedly, the rats acutely switched to the high‐phosphorus diet had even greater BBMV phosphorus uptake efficiency than the rats that continued the low‐phosphorus diet in a seemingly maladaptive response. Further, serum phosphorus was threefold higher after the acute high‐phosphorus load compared with rats kept on the low‐phosphorus diet, and twofold higher than rats that had been on the high‐phosphorus diet for 7 days in a separate experiment. These data suggest that dietary phosphorus restriction may cause an unintended adverse increase in phosphorus absorption efficiency and serum phosphorus after dietary non‐adherence. However, this has not been evaluated in rats with CKD, nor with in vivo intestinal phosphorus absorption testing methods.

The primary aim of this study was to test the hypothesis that a single bout of high‐phosphorus intake after acclimation to a low‐phosphorus diet in 5/6 nephrectomized male rats will increase intestinal phosphorus absorption. Secondary outcomes included plasma biochemistries related to CKD‐MBD and gene expression of the intestinal phosphate transporters, NaPi‐2b/*slc34a2*, sodium‐dependent phosphate co‐transporter 1 (PiT‐1/*slc20a1*), and sodium‐dependent phosphate co‐transporter 2 (PiT‐2/*slc20a2*). Based on Giral and colleagues,^(^ ^16^ ^)^ we hypothesized that intestinal fractional phosphorus absorption (efficiency) would be highest in rats acclimated to the low‐phosphorus diet then acutely switched to the high‐phosphorus diet.^(^ ^22^ ^)^

## Materials and Methods

### Study design

**Fig. 1:**
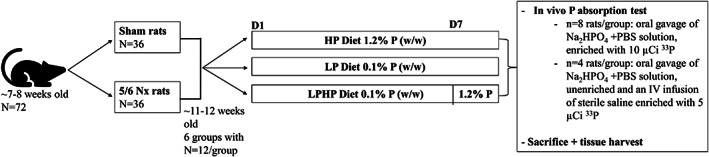
At ~7 to 8 weeks old, rats underwent either a two‐step 5/6 nephrectomy or sham operation. Four weeks post‐surgery (~11 to 12 weeks old), rats were switched to their randomly assigned diet treatment of either low phosphorus (LP; 0.1% w/w), high phosphorus (HP; 1.2% w/w), or low phosphorus followed by acute high phosphorus on the last day (LPHP; 0.1% w/w then 1.2% w/w). Rats were fed in a 4‐hour window (~8 a.m. to 12 p.m.) daily for 7 days and received water *ad libitum*. Rats in the LPHP group were fed the LP diet on days 1 to 6 and the HP diet on day 7.

In a 2 × 3 factorial design study, *n* = 72 commercial male Sprague Dawley rats (Charles River, Indianapolis, IN, USA) with 5/6 nephrectomy (*n* = 36) or sham operation (*n* = 36) were studied. Rats underwent a two‐step 5/6 nephrectomy surgery (at Charles River) at ~7–8 weeks of age and arrived at the study site at Purdue University (West Lafayette, IN, USA) approximately 1 week after completion of the second surgery. We received rats in four shipment cohorts of *n* = 18/shipment, which included *n* = 9 nephrectomized rats and *n* = 9 sham‐operated rats. Rats were block‐randomized to one of three dietary treatments within the four shipment cohort blocks and the two disease status blocks, with equal distribution of treatments in each shipment cohort and disease status block. This resulted in *n* = 12/group to one of three dietary treatment groups by disease status (nephrectomy or sham). Group allocation was concealed from investigators handling the rats only during the 3‐week acclimatization period, and the study was unblinded once the study diets and experimentation period began. Rats were housed on a 12‐hour light/dark cycle in a temperature‐ and humidity‐controlled room. Upon arrival to the study site, rats were group‐housed (2 rats/cage) in standard solid bottom caging with Aspen bedding for ~2 weeks and thereafter transferred to individual wire‐bottom metabolic cages 1 week before starting the diet treatment and through the end of the study. Before initiating study diets, rats were fed a non‐autoclaved grain and soy‐based standard rodent diet (Envigo Teklad 2018, Indianapolis, IN, USA) and received filtered water *ad libitum*. After the 3‐week acclimatization period (4 weeks post‐surgery and ~12 weeks of age), rats were switched to their randomly assigned diet treatment of either low phosphorus (LP; total P 0.1%, 0.6% Ca w/w, TD.85010 Envigo Teklad), high phosphorus (HP; total P 1.2%, 0.6% Ca w/w, TD.85349 Envigo Teklad), or low phosphorus followed by acute high phosphorus on the last (7th) day (LPHP, 0.1% w/w then 1.2% w/w). All study diets were non‐autoclaved egg‐white protein based (Supplemental Table S1). Access to food was restricted to a daily 4‐hour feeding window (~8 a.m. to 12 p.m.) for 7 days, with water *ad libitum*. LPHP rats were fed the LP diet on days 1 to 6 and the HP diet on day 7 (Fig. 1). The diet intervention duration, 4‐hour feeding window, and the low (0.1% w/w) and high (1.2% w/w) dietary phosphorus levels were chosen based on the study design of Giral and colleagues.^(^ ^16^ ^)^ After the 4‐hour feeding window on day 7, rats underwent an in vivo intestinal phosphorus absorption testing procedure (oral gavage test). Blood draws for biochemical analyses were taken at baseline (day 1, before start of study diet, jugular vein draw) and after euthanasia (day 7, abdominal aortic draw). Rats were weighed thrice weekly and food was weighed daily while rats were on the study diets to determine food consumption. This protocol was approved by the Purdue University Animal Care and Use Committee (protocol Nnumber: 1402001030). The study was pre‐registered at Animal Study Registry (10.17590/asr.0000207).

### Jugular catheter placement

Jugular catheters were placed using aseptic technique as a survival surgery 48 hours before intestinal phosphorus absorption efficiency testing. Once anesthetized, rats received pain medication of 2 mg/kg body weight Metacam NSAID injectable or 4–5 mg/kg body weight Carprofen. Rats were then placed in dorsal recumbency, the ventral cervical area shaved, sterilized, and a 2 cm incision was made in the skin over the right jugular vein. The fascia and underlying cervical muscles were separated by blunt dissection to reveal the jugular vein. The vein was isolated, cut, and the catheter was inserted. It was then threaded subcutaneously through the skin to the exit site, at the back of the neck between the shoulder blades. Upon determining patency, incision sites were sutured and rats were taken off anesthetic. After completion of surgery, rats were monitored for postoperative complications and catheters were flushed with heparinized saline every 12 hours to maintain patency.

### Intestinal phosphorus absorption efficiency

Intestinal phosphorus absorption efficiency was determined by in vivo oral gavage absorption testing performed before euthanasia. Absorption tests were conducted on rats in order of their randomization. After the 4‐hour feeding window on day 7, *n* = 8 rats/treatment group were orogastric gavaged with 3 mL of a transport solution enriched with 10 μCi ^33^P (^33^P‐orthophosphoric acid, American Radiolabeled Chemicals, Inc., St. Louis, MO, USA). The transport solution consisted of Na_2_HPO_4_ and phosphate‐buffered saline (PBS) standardized to contain 6.4 mg P for rats on the LP diet treatment and 76.8 mg P for rats on the HP and LPHP diet treatment (corresponding to ~1/3 of total daily P intake on each diet). To account for rate of renal clearance in calculating fractional intestinal phosphorus absorption, *n* = 4 rats/treatment group underwent an intravenous (iv) administration of 1 mL sterile saline enriched with 5 μCi ^33^P via jugular catheter. As the iv ^33^P was administered, these *n* = 4 rats/group were also given an oral gavage of unenriched Na_2_HPO_4_ and PBS transport solution containing either 6.4 mg P or 76.8 mg P according to their diet treatment group as described above (Fig. 1). Blood (0.25 mL/sampling) was collected by jugular catheters (placed 48 hours before phosphorus absorption testing) at 0, 20, 40, 60, 90, and 120 minutes after dosing with ^33^P.^(^ ^23^ ^)^ Blood was transferred to lithium heparin tubes, centrifuged at 10,000*g* for 10 minutes (Micro 18R, VWR, Radnor, PA, USA) for plasma. Liquid scintillation counting of plasma samples from each time point was performed on a Tri‐Carb 2910TR Liquid Scintillation Analyzer (PerkinElmer, Waltham, MA, USA). One hundred microliters of plasma was counted in 15 mL of EcoLite liquid scintillation cocktail (MP Biomedicals, Santa Ana, CA, USA). Intestinal fractional phosphorus absorption was determined from the ratio of area under the oral and iv plasma ^33^P curves (AUC_PO_/AUC_IV_), calculated for the *n* = 8 rats/group given the oral gavage ^33^P, using the AUC_IV_ average value from the *n* = 4 rats in the same group given the iv ^33^P.

### Tissue and blood collection

Rats were euthanized via CO_2_ asphyxiation immediately after the phosphorus absorption testing. After euthanasia, the abdominal cavity was opened, and a terminal blood draw was collected from the abdominal aorta and placed in lithium heparin tubes for separation of plasma. The small intestine was excised from the pyloric sphincter to the cecum at the ileo‐cecal junction. The excised intestine was flushed with sterile 0.9% NaCl to remove contents and was further cut into sections of duodenum (1 cm distal from the pyloric sphincter to ~10 cm) and jejunum (~10–30 cm). The mucosal layers of the duodenum (~10 cm) and jejunum (~10 cm) were scraped. Mucosal scrapings from each intestinal segment were flash‐frozen in liquid nitrogen and stored at −80°C for later mRNA extraction.

### Intestinal gene expression

Total RNA from duodenum and jejunum was isolated using miRNeasy Mini Kit (Qiagen, Valencia, CA, USA). Target‐specific PCR primers were obtained from Applied Biosystems (Foster City, CA, USA): NaPi2b (Slc34a2; Rn00584515_m1); PiT1 (Slc20a1; Rn00579811_m1); PiT2 (Slc20a2; Rn00568130_m1); ribosomal protein, large, P0 (RPLP0, Rn03302271_gH). The gene expression was determined by real‐time PCR using TaqMan gene expression assay system (TaqMan MGP probes, FAM dye‐labeled; Applied Biosystems) using ViiA 7 systems. The cycle number at which the amplification plot crosses the threshold was calculated (CT), and the ∆∆CT method was used to analyze the relative changes in mRNA expression and normalized by RPLP0.^(^ ^24^ ^)^

### Plasma biochemistries

Plasma was stored at −80°C and thawed before biochemical analyses. Plasma blood urea nitrogen (BUN), calcium, and phosphorus were determined by colorimetric assays (Point Scientific, Canton, MI, USA), iPTH and intact fibroblast growth factor‐23 (iFGF23) by enzyme‐linked immunosorbent assay (ELISA) (Quidel Corporation, San Diego, CA, USA), and 1,25D by enzyme immunoassay (EIA) (Immunodiagnostic Systems, The Boldons, UK).

### Statistics

Appropriate sample size was calculated based on phosphorus absorption data in 5/6 nephrectomized rats from Marks et al.^(^ ^25^ ^)^ Based on this, a sample size of *n* = 8 was deemed sufficient to detect a 30% difference between groups for intestinal phosphorus absorption efficiency (β = 0.80, α = 0.05). Statistical analysis was performed using Statistical Analysis Software (SAS) version 9.4 (SAS Institute, Cary, NC, USA). Two‐way ANCOVA was performed for all outcomes with cohort as a covariate (4 cohorts), main effects for health status (2 levels: CKD and sham), diet treatment (3 levels: LP, HP, LPHP), and diet treatment*health status (interaction). For oral and iv plasma curves, five sampling time points, all two‐way interactions, and the three‐way interaction of health status*diet treatment*time point were performed. Post hoc group comparisons with Tukey adjustments were made as appropriate based on the overall model findings. Statistical significance was set at α < 0.05.

As BUN level is a biomarker of kidney function, BUN values were inspected for all rats. Sham rats with abnormal BUN values were suspected to be due to potential kidney injury during sham operation and were removed if they had a studentized deleted residual (SDR) ≥2.7. Outliers in BUN (*n* = 5, 3 sham HP, 1 sham LP, and 1 CKD HP) were removed from all other analyses. *n* = 1 rat (sham, LPHP) died at ~12 weeks of age, during the jugular catheter placement surgery despite no apparent complications; an enlarged stomach with compacted intestines were found upon necropsy. Thus, *n* = 66 rats were included for analyses of all outcomes of interest. For each outcome, outliers were investigated and removed if SDR was ≥3. Body weight and day‐7 food intake were explored as additional covariates for intestinal fractional phosphorus absorption, but these were not significant and, therefore, not included. Appropriate data transformations were performed for variables with non‐normal distribution of residuals as determined by Shapiro–Wilk tests. Log‐transformation was used for iFGF23, jejunal NaPi‐2b, jejunal PiT1, and jejunal PiT2, and square‐root transformation was used for analysis of duodenal NaPi‐2b and PiT1. These variables were back‐transformed and presented in the original units in the results. Results are reported as least‐squares mean ± SD, unless otherwise indicated.

## Results

During the 7‐day diet study, rats progressively increased their daily food consumption in the 4‐hour feeding window, with an average of 17.9 ± 4.42 g consumed on the final day. No difference was observed among groups for food consumption on day 7 (*p* = 0.25) (Supplemental Table S2). On day 7, sham rats had greater body weight compared with CKD rats (371 ± 27.3 g and 340 ± 27.2 g, respectively, *p* < 0.0001). There was no difference in body weight by diet treatment nor an interaction effect (Supplemental Table S2). As expected, the final plasma BUN was higher in CKD rats compared with sham rats (*p* < 0.0001) (Table 1).

**Table 1:** Intestinal Fractional Phosphorus Absorption and Endpoint Plasma Biochemistries

	Health status × diet						ANCOVA *p* values			
	LP		LPHP		HP					
	Sham	CKD	Sham	CKD	Sham	CKD	Model	Health status	Diet	H × D
Intestinal fractional phosphorus absorption (AUC_PO_/AUC_IV_)^a^	0.13 (0.05)	0.14 (0.05)	0.08 (0.05)	0.10 (0.06)	0.08 (0.05)	0.10 (0.06)	0.12	0.31	**0.03**	0.97
BUN (mg/dL)^b^	23.3 (5.11)	40.3 (5.09)	21.9 (5.11)	39.3 (5.09)	22.2 (5.16)	37.2 (5.11)	**<0.0001**	**<0.0001**	0.40	0.73
P (mg/dL)^c^	6.3 (1.13)^c^	5.2 (1.14)^c^	11.4 (1.13)^b^	13.5 (1.13)^a^	11.4 (1.14)^b^	12.5 (1.14)^a^, ^b^	**<0.0001**	**0.02**	**<0.0001**	**<0.0001**
Ca (mg/dL)^d^	11.3 (1.16)	11.4 (1.14)	10.3 (1.14)	9.3 (1.14)	10.7 (1.14)	11.2 (1.16)	**<0.0001**	0.71	**0.0001**	0.08
iFGF23 (pg/mL)^e^	126 (46.1)^c^	183 (63.4)^c^	340 (118.6)^b^	420 (145.8)^b^	327 (117.9)^b^	837 (302.1)^a^	**<0.0001**	**<0.0001**	**<0.0001**	**0.006**
iPTH (pg/dL)^f^	820 (633.8)	594 (632.5)	1559 (633.8)	1114 (632.5)	1990 (637.5)	2358 (633.8)	**<0.0001**	0.53	**<0.0001**	0.12
1,25(OH)_2_D_3_ (pg/mL)^g^	143 (50.4)	133 (50.1)	157 (50.3)	142 (49.9)	142 (50.4)	161 (50.1)	0.53	0.87	0.68	0.51

LP = low‐phosphorus diet; LPHP = low‐phosphorus diet for 6 days followed by acute high‐phosphorus on day 7; HP = high‐phosphorus diet; CKD = chronic kidney disease; AUC_PO_/AUC_IV_ = ratio of area under the oral and iv plasma ^33^P curves; BUN = blood urea nitrogen; P = phosphorus; Ca = calcium; iFGF23 = intact fibroblast growth factor‐23; iPTH = intact parathyroid hormone; 1,25(OH)_2_D_3_ =1alpha, 25‐dihydroxyvitamin D3. ANCOVA *p* values for the overall model (*P*_Model_), main of effect of health status (*P*_Health_), diet (*P*_Diet_), and their interaction (*P*_HxD_) for intestinal fractional phosphorus absorption and plasma biochemistries. Least‐squares (LS) means ± SD are shown. Intestinal fractional phosphorus absorption was not different among groups. Plasma BUN was higher in CKD rats compared with sham. Plasma phosphorus was higher in HP and LPHP groups compared with the LP group regardless of health status and was higher in CKD rats in the LPHP group compared with sham rats in the LPHP group. Plasma calcium was lower in the LPHP group compared with rats in the HP and LP group. Plasma iFGF23 was lowest in rats in the LP group and was highest in CKD rats in the HP group. Plasma iPTH was lowest in rats in the LP group followed by rats in the LPHP group and highest in rats in the HP group. Plasma 1,25D did not differ among groups. Different superscripted letters represent group differences. Significant *p* values are indicated in bold font.

^a^ *n* = 7 sham LP, 7 sham LPHP, 6 sham HP, 7 CKD LP, 8 CKD LPHP, 8 CKD HP.

^b^ *n* = 11 sham LP, 11 sham LPHP, 9 sham HP, 12 CKD LP, 12 CKD LPHP, 11 CKD HP.

^c^ *n* = 11 sham LP, 11 sham LPHP, 9 sham HP, 12 CKD LP, 11 CKD LPHP, 10 CKD HP.

^d^ *n* = 11 sham LP, 10 sham LPHP, 9 sham HP, 12 CKD LP, 12 CKD LPHP, 11 CKD HP.

^e^ *n* = 11 sham LP, 10 sham LPHP, 9 sham HP, 12 CKD LP, 12 CKD LPHP, 9 CKD HP.

^f^ *n* = 11 sham LP, 11 sham LPHP, 8 sham HP, 12 CKD LP, 12 CKD LPHP, 11 CKD HP.

^g^ *n* = 9 sham LP, 10 sham LPHP, 9 sham HP, 11 CKD LP, 12 CKD LPHP, 11 CKD HP.

**Fig. 2:**
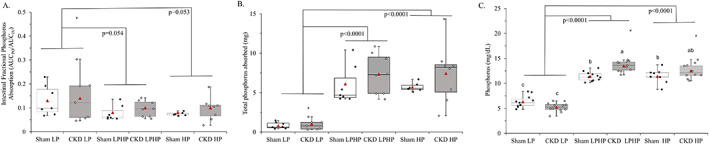
(*A*) Intestinal fractional phosphorus absorption by health status and dietary phosphorus load. Intestinal fractional phosphorus absorption was determined from the ratio of area under the oral and iv plasma ^33^P curves (AUC_PO_/AUC_IV_) over a 2‐hour period. Intestinal fractional phosphorus absorption was not significant in the overall model (*P*_Model_ = 0.12, *P*_Health_ = 0.31, *P*_Diet_ = 0.03, *P*_HxD_ = 0.97). (*B*) Absolute amount of phosphorus absorbed by health status and dietary phosphorus load. Rats in the high‐phosphorus diet (HP) and low‐phosphorus diet for 6 days followed by acute high‐phosphorus on day 7 (LPHP) groups absorbed a significantly greater amount of the dose compared with the low‐phosphorus diet (LP) group (*P*_Model_ <0.0001, *P*_Health_ = 0.12, *P*_Diet_ <0.0001, *P*_HxD_ 0.65). (*C*) Plasma phosphorus by health status and dietary phosphorus load. Chronic kidney disease (CKD) and sham rats in the LPHP and HP groups had higher plasma phosphorus than rats in the LP group. Within diets, CKD rats had higher plasma phosphorus compared with sham only in the LPHP group (*P*_Model_ <0.0001, *P*_Health_ = 0.02, *P*_Diet_ <0.0001, *P*_HxD_ <0.0001). The median, interquartile range, minimum, and maximum, including outliers, are shown. X symbols denote outliers not included in ANCOVA. Least‐squares (LS) means are represented by triangles for each group. Sham rats are shown with white boxes, and CKD rats are shown with gray boxes. Fractional phosphorus absorption and amount of dose absorbed *n* = 7 sham LP, 7 sham LPHP, 6 sham HP, 7 CKD LP, 8 CKD LPHP, 8 CKD HP. Plasma phosphorus *n* = 11 sham LP, 11 sham LPHP, 9 sham HP, 12 CKD LP, 12 CKD LPHP, 11 CKD HP.

**Fig. 3:**
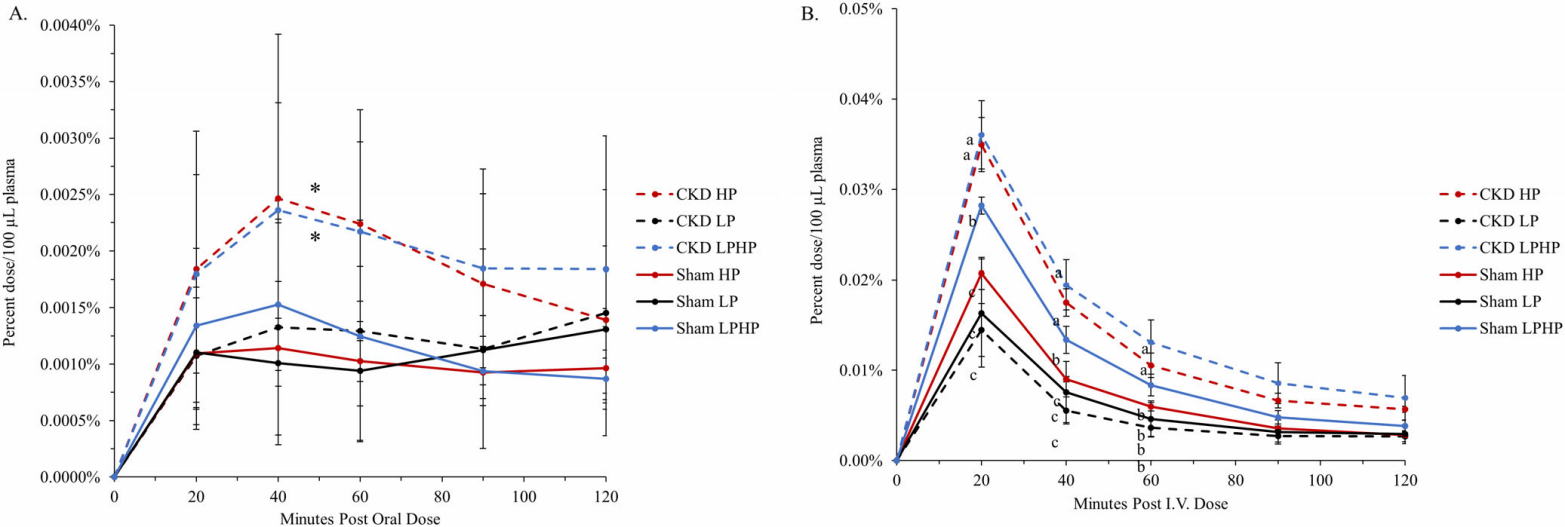
(*A*) Average oral dose curves. Group mean plasma ^33^P levels at each time point are shown as percent of oral isotope dose with SD error bars. No difference was observed for the interaction of health status by diet treatment by time point over the 2‐hour period (*p* = 0.98). There was significant two‐way interaction between diet treatment and health status (*p* = 0.03). *mean plasma ^33^P as percent of oral isotope dose was significantly higher in the chronic kidney disease (CKD) high‐phosphorus diet (HP) and CKD low‐phosphorus diet for 6 days followed by acute high‐phosphorus on day 7 (LPHP) groups compared with all other group means over the 120‐minute period (*p* < 0.05), but there were no significant differences at any individual time point. (*B*) Average iv dose curves. Group mean plasma ^33^P levels at each time point are shown as percent of iv isotope dose with SD error bars. There was a significant three‐way interaction of health status by diet treatment by time point (*p* = 0.007). Post hoc comparisons of group means within each time point were evaluated. Within each time point, group means that are significantly different are indicated by different letters (*p* < 0.05). Mean plasma 33P as percent of iv isotope dose was significantly higher in the CKD HP and CKD LPHP groups at 20, 40, and 60 minutes compared with all other groups. Additionally, the sham LPHP group had higher plasma 33P compared with the sham low‐phosphorus diet (LP), sham HP diets CKD LP groups at 20 and 40 minutes. By 90 and 120 minutes, there were no differences between groups. Values presented are from 100 μL of plasma counted at each time point over a 2‐hour period. Average oral dose curves: *n* = 7 sham LP, 7 sham LPHP, 6 sham HP, 7 CKD LP, 8 CKD LPHP, 8 CKD HP. Average iv dose curves: *n* = 4 sham LP, 4 sham LPHP, 3 sham HP, 4 CKD LP, 4 CKD LPHP, 3 CKD HP.

Intestinal fractional phosphorus absorption was not significant in the overall model (*p* = 0.12) (Table 1). However, exploratory comparisons showed intestinal fractional phosphorus absorption efficiency tended to be higher in rats in the LP group compared with rats in the HP group (*P*(diff) = 0.053) or LPHP group (*P*(diff) = 0.054) (Table 1; Fig. 2*A*; Supplemental Table S3), whereas HP and LPHP groups were similar (*P*(diff) = 0.998). The absolute amount of dose absorbed was significantly higher in the HP (6.6 ± 2.2 mg P) and LPHP (6.7 ± 2.2 mg P) compared with the LP group (0.9 ± 2.2 mg P) (*P*(diff) < 0.0001 for both diet comparisons) (Fig. 2*B*). Additional detail on the oral and iv dose curves are shown in Fig. 3 and individual rat curves are provided in Supplemental Figs. S1 and S2.

**Fig. 4:**
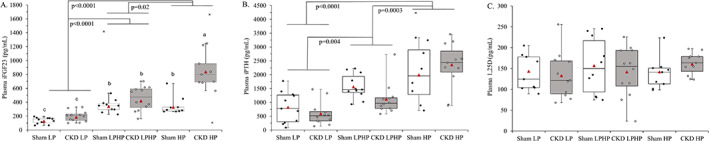
(*A*) Plasma iFGF23 by health status and dietary phosphorus load. Plasma iFGF23 was lowest in rats in the low‐phosphorus diet (LP) group regardless of health status and was ~2× higher in chronic kidney disease (CKD) rats in the high‐phosphorus diet (HP) group compared with any other group (*P*_Model_ <0.0001, *P*_Health_ <0.0001, *P*_Diet_ <0.0001, *P*_HxD_ = 0.006). (*B*) Plasma iPTH by health status and dietary phosphorus load. Plasma phosphorus was lowest in rats in the LP group, followed by the low‐phosphorus diet for 6 days followed by acute high‐phosphorus on day 7 (LPHP) group, and highest in the HP group (*P*_Model_ <0.0001, *P*_Health_ = 0.53, *P*_Diet_ <0.0001, *P*_HxD_ = 0.12). (*C*) Plasma 1,25D by health status and dietary phosphorus load. Plasma 1,25D did not differ between groups (*P*_Model_ = 0.53, *P*_Health_ = 0.87, *P*_Diet_ = 0.68, *P*_HxD_ = 0.51). The median, interquartile range, minimum, and maximum, including outliers, are shown. X symbols denote outliers not included in ANCOVA. Least‐squares (LS) means are represented by triangles for each group. Sham rats are shown with white boxes and CKD rats are shown with gray boxes. Plasma iFGF23 and iPTH *n* = 11 sham LP, 11 sham LPHP, 9 sham HP, 12 CKD LP, 12 CKD LPHP, 11 CKD HP. Plasma 1,25D *n* = 9 sham LP, 10 sham LPHP, 9 sham HP, 11 CKD LP, 12 CKD LPHP, 11 CKD HP.

For plasma phosphorus, there was a significant health status by diet interaction (*p* < 0.0001): Plasma phosphorus was not different between CKD and sham rats with the LP or HP diets, but with the LPHP diet, CKD rats had higher plasma phosphorus than sham rats (*P*(diff) = 0.001). Regardless of health status, plasma phosphorus was higher with HP and LPHP diets compared with the LP diet (*P*(diff) < 0.0001 for both comparisons, respectively) (Table 1; Fig. 2*C*; Supplemental Table S3).There was a significant main effect for diet on plasma calcium (*p* < 0.0001): Plasma calcium values were significantly lower in rats in the LPHP group compared with rats in the HP group (*P*(diff) = 0.006) and LP group (*P*(diff) < 0.0001) but were not different between the HP and LP groups (*P*(diff) = 0.56) (Table 1; Supplemental Table S3).There was a significant health status by diet interaction for plasma iFGF23 (*p* = 0.006). Plasma iFGF23 was lowest in rats in the LP group compared with the HP and LPHP groups (*P*(diff) < 0.0001 for both comparisons, respectively), but there was only an effect of CKD within the HP group where plasma iFGF23 was ~2× higher in CKD rats compared with sham (*P*(diff) <0.0001) (Table 1; Fig. 4*A*; Supplemental Table S3). There was a significant main effect for diet on plasma iPTH (*p* < 0.0001), which was lowest in the LP group, followed by LPHP group, and highest in the HP group (*P*(diff) < 0.01 for all diet comparisons) (Table 1; Fig. 4*B*; Supplemental Table S3). The overall two‐way ANCOVA model for 1,25D was not significant (*p* = 0.53) (Table 1; Fig. 4*C*; Supplemental Table S3).

**Fig. 5:**
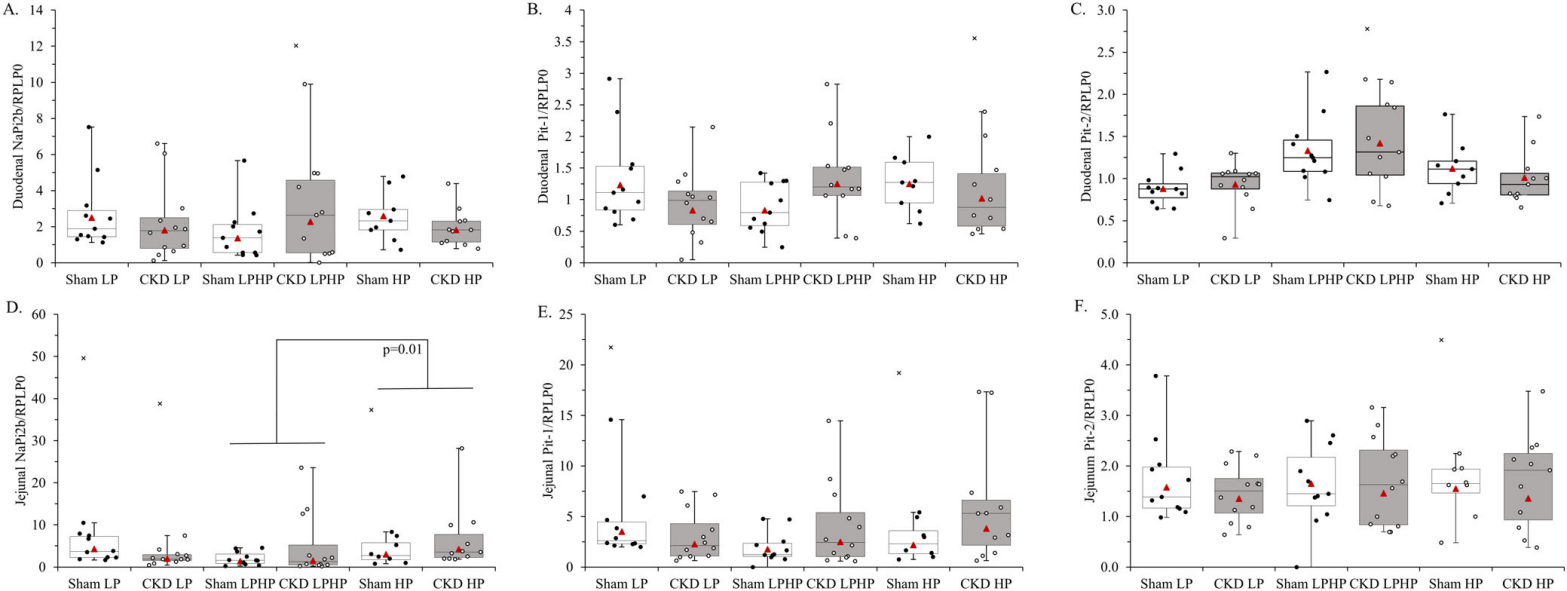
(*A*) Duodenal NaPi‐2b mRNA expression by health status and dietary phosphorus load. Duodenal NaPi‐2b mRNA expression did not differ between groups (*P*_Model_ = 0.27, *P*_Health_ = 0.72, *P*_Diet_ = 0.71, *P*_HxD_ = 0.17). (*B*) Duodenal PiT‐1 mRNA expression by health status and dietary phosphorus load. Duodenal PiT‐1 mRNA expression did not differ between groups (*P*_Model_ = 0.49, *P*_Health_ = 0.66, *P*_Diet_ = 0.82, *P*_HxD_ = 0.0501). (*C*) Duodenal PiT‐2 mRNA expression by health status and dietary phosphorus load. Duodenal PiT‐2 mRNA expression was higher in rats in the low‐phosphorus diet for 6 days followed by acute high‐phosphorus on day 7 (LPHP) group compared with rats in the low‐phosphorus diet (LP) and high‐phosphorus diet (HP) groups (*P*_Model_ = 0.01, *P*_Health_ = 0.91, *P*_Diet_ = 0.0002, *P*_HxD_ = 0.64). (*D*) Jejunal NaPi‐2b mRNA expression by health status and dietary phosphorus load. Jejunal NaPi‐2b mRNA expression was lower in rats in the LPHP group compared with rats in the HP group and tended to be lower than rats in the LP group (*P*_Model_ = 0.007, *P*_Health_ = 0.69, *P*_Diet_ = 0.01, *P*_HxD_ = 0.18). (*E*) Jejunal PiT‐1 mRNA expression by health status and dietary phosphorus load. Jejunal PiT‐1 mRNA expression did not differ between groups (*P*_Model_ = 0.33, *P*_Health_ = 0.47, *P*_Diet_ = 0.41, *P*_HxD_ = 0.16). (*F*) Jejunal PiT2 mRNA expression by health status and dietary phosphorus load. Jejunal PiT‐2 mRNA expression did not differ between groups (*P*_Model_ = 0.0003, *P*_Health_ = 0.19, *P*_Diet_ = 0.86, *P*_HxD_ = 0.98). The significance in the *P*_Model_ is due to a cohort effect rather than main effects for health status, diet, or their interaction. Expression was calculated relative to Rplp0. The median, interquartile range, minimum, and maximum, including outliers, are shown. X symbols denote outliers not included in the ANCOVA. Least squares (LS) means are represented by triangles for each group. Sham rats are shown with white boxes and CKD rats are shown with gray boxes. Duodenal NaPi‐2b, duodenal PiT1, and duodenal PiT2 *n* = 11 sham LP, 11 sham LPHP, 9 sham HP, 12 CKD LP, 12 CKD LPHP, 11 CKD HP. Jejunal Napi‐2b, jejunal PiT1, and jejunal PiT2 *n* = 11 sham LP, 11 sham LPHP, 9 sham HP, 12 CKD LP, 12 CKD LPHP, 11 CKD HP.

Intestinal phosphate transporters NaPi‐2b, PiT‐1, and PiT‐2 were evaluated for changes in gene expression in the duodenum and jejunum. The overall two‐way ANCOVA model for duodenal NaPi‐2b and duodenal PiT‐1 were not significant (*p* = 0.27 and *p* = 0.49, respectively) (Table 2; Fig. 5*A*, *B*; Supplemental Table S4). There was a significant diet effect for duodenal PiT‐2 (*p* = 0.0002). Duodenal PiT‐2 mRNA expression was higher in rats in the LPHP group compared with rats in the LP and HP groups (*P*(diff) = 0.0002 and *P*(diff) = 0.02) (Table 2; Fig. 5*C*; Supplemental Table S4). There was a significant diet effect for jejunal NaPi‐2b (*p* = 0.01), where it was lower in rats in the LPHP group compared with rats in the HP group and tended to be lower than rats in the LP group (*P*(diff) = 0.01 and *P*(diff) = 0.06) (Table 2; Fig. 5*D*; Supplemental Table S4). The overall two‐way ANCOVA model for jejunal PiT‐1 was not significant (*p* = 0.33) (Table 2; Fig. 5*E*; Supplemental Table S4). For jejunal PiT‐2, the overall ANCOVA model was significant (*p* = 0.0003), but this was only due to the significant covariate (cohort), as there were no significant main effects or interaction (Table 2; Fig. 5*F*; Supplemental Table S4).

**Table 2:** Intestinal Phosphate Transporters Gene Expression

	Health status × diet						ANCOVA *p* values			
	LP		LPHP		HP					
	Sham	CKD	Sham	CKD	Sham	CKD	Model	Health status	Diet	H × D
Duodenal NaPi‐2b/RPLP0^a^	2.50 (1.89)	1.80 (1.59)	1.37 (1.39)	2.28 (1.79)	2.59 (1.92)	1.82 (1.63)	0.27	0.72	0.71	0.17
Duodenal Pit‐1/RPLP0^b^	1.23 (0.66)	0.83 (0.52)	0.83 (0.53)	1.25 (0.62)	1.25 (0.66)	1.02 (0.57)	0.49	0.66	0.82	0.0501
Duodenal Pit‐2/RPLP0^a^	0.88 (0.36)	0.93 (0.35)	1.33 (0.36)	1.42 (0.36)	1.12 (0.36)	1.01 (0.36)	**0.01**	0.91	**0.0002**	0.64
Jejunal NaPi‐2b/RPLP0^c^	4.26 (4.24)	1.99 (2.02)	1.36 (1.39)	1.55 (1.52)	3.03 (3.05)	4.22 (4.28)	**0.007**	0.69	**0.01**	0.18
Jejunal Pit‐1/RPLP0^d^	3.49 (3)	2.27 (1.97)	1.77 (1.59)	2.48 (2.18)	2.18 (1.95)	3.82 (3.32)	0.33	0.47	0.41	0.16
Jejunum Pit‐2/RPLP0^e^	1.58 (0.63)	1.35 (0.55)	1.65 (0.70)	1.46 (0.62)	1.55 (0.65)	1.36 (0.53)	**0.0003**	0.19	0.86	0.98

LP = low‐phosphorus diet; LPHP = low‐phosphorus diet for 6 days followed by acute high‐phosphorus on day 7; HP = high‐phosphorus diet; CKD = chronic kidney disease; NaPi‐2b= sodium‐dependent phosphate transport protein 2B; RPLP0 = ribosomal protein, large, P0; Pit‐1 = sodium‐dependent phosphate co‐transporter 1; Pit‐2 = sodium‐dependent phosphate co‐transporter 2. ANCOVA *p* values for the overall model (*P*_Model_), main of effect of health status (*P*_Health_), diet (*P*_Diet_), and their interaction (*P*_HxD_) for the intestinal phosphate transporters NaPi‐2b, PiT‐1, and PiT‐2. Least‐squares (LS) means ± SD are shown. Duodenal NaPi‐2b and PiT‐1 mRNA expression did not differ among groups. Duodenal PiT‐2 mRNA expression was higher in rats in the LPHP group compared with rats in the LP and HP groups. Jejunal NaPi‐2b mRNA expression was lower in rats in the LPHP group compared with rats in the HP group and tended to be lower than rats in the LP group. Jejunal PiT‐1 and PiT‐2 mRNA expression did not differ among groups. Significant *p* values are indicated in bold font.

^a^ *n* = 11 sham LP, 11 sham LPHP, 9 sham HP, 12 CKD LP, 11 CKD LPHP, 11 CKD HP.

^b^ *n* = 11 sham LP, 11 sham LPHP, 9 sham HP, 12 CKD LP, 12 CKD LPHP, 10 CKD HP.

^c^ *n* = 10 sham LP, 11 sham LPHP, 8 sham HP, 11 CKD LP, 12 CKD LPHP, 11 CKD HP.

^d^ *n* = 10 sham LP, 10 sham LPHP, 8 sham HP, 12 CKD LP, 12 CKD LPHP, 11 CKD HP.

^e^ *n* = 11 sham LP, 10 sham LPHP, 8 sham HP, 12 CKD LP, 12 CKD LPHP, 11 CKD HP.

## Discussion

In the present study, we found that acute high‐phosphorus intake after a 6‐day acclimation to a low phosphorus diet in 5/6 nephrectomized and sham‐operated rats did not enhance intestinal fractional phosphorus absorption efficiency in vivo. This is supported by the lower (rather than higher) jejunal NaPi‐2b mRNA expression observed in the LPHP group compared with the HP group. Despite this, the acute high‐phosphorus load in the LPHP group led to higher plasma phosphorus compared with the LP group but similar plasma phosphorus to that of the HP group. This was contrary to our hypothesis and the previous findings of Giral and colleagues,^(^ ^16^ ^)^ where plasma phosphorus was ~2 times higher in healthy rats fed an acute high‐phosphorus load after acclimation to a low‐phosphorus diet than rats only fed the high‐phosphorus diet chronically for all 7 days (8.3 ± 0.9 mg/dL versus 17.2 ± 1.8 mg/dL). However, they studied the chronic high‐phosphorus diet and the low‐to‐acute high‐phosphorus diet in two separate experiments with slightly different feeding protocols (*ad libitum* versus 4‐hour feeding window), which limits the comparison between these two treatments. In our study, we also observed a lower plasma Ca in the LPHP group but similar plasma Ca between the LP and HP groups. This could possibly be due to the rapid increase in phosphorus intake in LPHP rats when switched from the LP to HP diet and an abrupt binding of dietary Ca leading to a transient decrease in serum Ca from lower Ca absorption.

In our study, CKD rats had higher iFGF23 compared with sham rats only when fed the HP diet for 7 days but was not different between CKD and sham rats in the LP group nor in the LPHP group that were both fed the low‐phosphorus diet for the first 6 days and only differed when the LPHP group was given the high‐phosphorus load on day 7. However, the acute high‐phosphorus load resulted in higher iFGF23 in both CKD and sham rats compared with CKD and sham rats kept on the low‐phosphorus diet. Others have observed increased iFGF23 in response to high phosphorus intakes^(^ ^26^, ^27^, ^28^, ^29^ ^)^ in humans, in line with its role as the major known phosphaturic hormone. But this has not been observed over a short time frame of only 4 hours;^(^ ^30^ ^)^ rather, increases in iFGF23 have been observed as a delayed response after ~24 hours.^(^ ^26^, ^27^, ^28^ ^)^ In our study, FGF23 was elevated within 6 hours of acute high phosphorus in the LPHP group. A possible explanation for this relatively quick response is that the acute high‐phosphorus diet was 10‐fold higher in phosphorus content compared with the low‐phosphorus diet on which these rats were acclimated. This is consistent with the findings of Nishida and colleagues,^(^ ^31^ ^)^ where FGF23 was measured at 4 and 8 hours after test meals of 400, 800, and 1200 mg P loads in non‐CKD adults. FGF23 was only elevated at the 8‐hour time point with the very high P load of 1200 mg. This indicates that higher dietary phosphorus may be required to observe a response in FGF23 at earlier time points. Notably, sham rats in the HP group fed the high‐phosphorus diet for all 7 days did not have higher iFGF23 than those in the LPHP group that only had 1 day of acute high phosphorus. Conversely, CKD rats fed the HP diet for 7 days had significantly higher plasma iFGF23 compared with those in the LPHP group, indicating that CKD rats may have a lower tolerance for sustained excess phosphorus.

There were no differences between CKD and sham rats for plasma 1,25D or iPTH. This is consistent with these hormones typically changing later in the disease progression compared with iFGF23, which is elevated as early as stage 2 CKD.^(^ ^30^, ^32^ ^)^ Although numerous studies^(^ ^22^, ^25^, ^33^ ^)^ have observed lower 1,25D in CKD rat models compared with controls, others^(^ ^34^ ^)^ have reported no difference between 5/6 nephrectomized and control rats in a 9‐week study. The diet effects on plasma iPTH, where the LP groups had the lowest values, followed by LPHP and highest with HP, are consistent with the expected effect on PTH as a phosphaturic hormone, increasing with higher dietary phosphorus exposure. Particularly, the higher iPTH in the LPHP group compared with the LP group is consistent with the observed role of PTH as an acute responder to alterations in dietary phosphorus intake.^(^ ^31^, ^35^, ^36^ ^)^ Martin and colleagues^(^ ^37^ ^)^ found that PTH increased as much as 80% within only 15 minutes of high phosphorus consumption in uremic rats.

The lack of enhanced phosphorus absorption efficiency in the LPHP group is contrary to previous findings in healthy rats that assessed intestinal phosphate uptake by in vitro methods.^(^ ^16^ ^)^ Giral and colleagues^(^ ^16^ ^)^ reported a fivefold higher sodium‐dependent uptake of phosphate in BBMV isolated from the duodenum of rats fed an acute high‐phosphorus load after acclimation to a low‐phosphorus diet. The differences in these findings may be attributable, at least in part, to the difference between the in vivo and in vitro techniques used to assess absorption efficiency in each study, in addition to the different rat models. The BBMV rapid filtration approach measures radioactive phosphorus uptake into BBMV isolated from intestinal mucosal scrapings, and thus assesses transport of phosphate across the apical membrane but lacks the intracellular and basolateral membrane components of the cell. In contrast, the in vivo oral gavage intestinal absorption method used in this study keeps the intestinal epithelium intact and physiological systems in place.^(^ ^38^ ^)^ Our results suggest that the enhanced in vitro phosphate uptake into isolated duodenal BBMV after an acute phosphorus load may not translate to enhanced in vivo intestinal phosphorus absorption efficiency along the entirety of the gastrointestinal tract. This adds to the few but growing number of in vivo intestinal phosphorus absorption studies contradicting previous findings from ex vivo methods of intestinal phosphate uptake. Although ex vivo/in vitro studies have repeatedly shown higher intestinal phosphate uptake efficiency induced by low‐phosphorus diets,^(^ ^16^, ^17^, ^18^, ^19^, ^20^, ^39^, ^40^, ^41^ ^)^ our group demonstrated that a low‐phosphorus diet (0.1% P w/w) did not affect intestinal phosphorus absorption efficiency in healthy male Sprague Dawley rats using a jejunal in situ ligated loop technique in live animals.^(^ ^42^ ^)^ Marks and colleagues^(^ ^25^ ^)^ reported similar results using the same low‐phosphorus diet and jejunal ligated loop method for assessing intestinal phosphorus absorption. In the current study, using the in vivo oral gavage test, we found only a non‐significant trend toward enhanced intestinal phosphorus absorption efficiency with the LP diet.

With declining kidney function, rising FGF‐23, and declining 1,25D, one would predict that intestinal phosphorus absorption efficiency would be suppressed. This is supported by studies showing that calcitriol increases expression of NaPi‐2b and intestinal phosphorus uptake or transport as assessed by in vitro and ex vivo methods in rodent models, ^(^ ^18^, ^20^, ^43^, ^44^, ^45^, ^46^ ^)^ and conversely, vitamin D‐receptor knockout mice have lower expression of NaPi‐2b.^(^ ^47^ ^)^ However, in vivo absorption studies have shown a general lack of effect of kidney disease on intestinal phosphorus absorption efficiency. In the present study, this could be explained by the lack of reduced 1,25D in the CKD rats. However, lack of effect of kidney disease on in vivo intestinal phosphorus absorption efficiency has been demonstrated in three different rat models of CKD with decreased 1,25D: Marks and colleagues^(^ ^25^ ^)^ found no difference in intestinal phosphorus absorption efficiency measured by the jejunal ligated loop method in 5/6 nephrectomized rats compared with sham. Turner and colleagues^(^ ^23^ ^)^ reported similar results in an adenine‐induced CKD rat model. And our group observed significantly *higher* intestinal phosphorus absorption efficiency in the Cy/+ rat model of progressive CKD‐MBD compared with normal littermates, but the difference was only slight.^(^ ^22^ ^)^ We also found that fractional intestinal phosphorus absorption, assessed by oral and iv administration of ^33^P, was not different between patients with stage 3–4 CKD and healthy controls, despite lower 1,25D observed in the patients with CKD.^(^ ^48^ ^)^ Thus, a compensatory response to decrease intestinal phosphorus absorption efficiency with declining kidney function appears to be lacking, at least in the moderate stages of disease progression. However, studies using in vivo intestinal phosphorus absorption methods are still relatively scarce; thus, more evidence is needed to draw firm conclusions regarding the discrepancies shown between the many in vitro studies and the few in vivo studies.

A rapid downregulation of jejunal NaPi‐2b gene expression was observed for rats in the LPHP group compared with the HP group, which may partly explain the lack of enhanced intestinal fractional phosphorus absorption in the LPHP group compared with the enhanced uptake in BBMV of healthy male rats.^(^ ^16^ ^)^ Similarly, Candeal and colleagues^(^ ^17^ ^)^ observed lower duodenal and jejunal NaPi‐2b and PiT‐1 protein expression after acute high‐phosphorus intake compared with low‐phosphorus intake. However, the fractional absorption test methods used in this study give total fractional phosphorus absorption that includes both the active transcellular and passive paracellular absorption pathways. In vivo, transcellular phosphate absorption has been shown to account for ~1/3 of total phosphorus absorption.^(^ ^22^, ^49^ ^)^ Thus, paracellular phosphorus absorption likely contributes to the similar plasma phosphorus values between the LPHP and HP groups in this study. However, future studies are required to confirm this.

Fluctuating from a low‐phosphorus intake to an acute high‐phosphorus intake, as is often the case with CKD patients, does not subsequently enhance fractional intestinal phosphorus absorption, but does lead to higher absolute phosphorus absorption and plasma phosphorus, according to our results. It also remains possible that other adverse consequences to such fluctuations may still occur. Indeed, Tani and colleagues^(^ ^50^ ^)^ observed higher vascular calcification in unilateral nephrectomized male rats when given a diet fluctuating every 2 days from a low‐phosphorus (0.02% P) to high‐phosphorus (1.2% P) diet over a 36‐day period (averaging 0.6% P) compared with rats fed a consistent phosphorus diet of 0.6% P. The rats fed the fluctuating diet had similar vascular calcification compared with rats fed a consistent high‐phosphorus diet (1.2% P), despite the latter group consuming twice as much phosphorus over the course of the 36‐day study. Similarly, Zelt and colleagues^(^ ^51^ ^)^ observed greater phosphate and calcium accumulation in the vasculature after an acute iv phosphate pulse in male rats with adenine‐induced CKD, suggesting that acute spikes in plasma phosphate may drive vascular calcification. This highlights the necessity of future studies investigating the effect of acute phosphorus intake after acclimation to a low‐phosphorus diet on cardiovascular endpoints in CKD.

One limitation of the current study was that intestinal phosphate transporter analysis was limited to mRNA expression, which may not accurately reflect changes in protein expression or location within the cytoplasm or brush border membrane. In addition, only a single bout of non‐adherence to the low‐phosphorus diet was tested and may not represent intestinal fractional phosphorus absorption after multiple bouts of non‐adherence or effects of the diets over a longer duration. Phosphorus absorption measurement was limited to a 2‐hour period, following the 4‐hour feeding window. Therefore, we were unable to determine if enhanced phosphorus absorption occurred at an earlier time during this 4‐hour window. If enhanced absorption took place, it is possible that compensation for the acute high‐phosphorus diet occurred by the time the phosphorus absorption test was performed. Further, the 2‐hour absorption period only accounts for phosphorus absorption in the small intestine rather than the entirety of the gastrointestinal tract, which has been observed to take ~6 hours.^(^ ^52^ ^)^ It is thought that the majority of phosphorus absorption occurs in the small intestine, although this remains uncertain.^(^ ^53^ ^)^

The low‐phosphorus diet level of 0.1% P w/w was chosen as consistent with low P diets commonly used in the rodent literature. However, this is only ~1/3 of the rat P requirement of 0.3% P w/w. Translating this to human intakes, this would be only ~210 mg/d P compared with the 700 mg/d P Recommended Dietary Allowance for adults.^(^ ^54^ ^)^ Similarly, the 1.2% high‐P diet is 4× the rat requirement, which would translate to a human intake of ~2800 mg/d, which is higher than the estimated average US intake (~1500 mg/d)^(^ ^13^ ^)^ but still below the current Tolerable Upper Intake Level as set by National Academy of Medicine.^(^ ^54^ ^)^ Thus, the difference in phosphorus intake between the LP and HP diets in this study is more drastic than what might typically be found in humans switching from an unrestricted to a restricted diet. Yet, even with this relatively extreme dietary challenge, no effects on fractional phosphorus absorption were observed. Differences in the main calcium sources in the two diets also existed, where the LP diet had calcium carbonate as the main calcium source, but the HP diet had calcium phosphate. Calcium carbonate would have a more metabolically alkaline effect compared with calcium phosphate, which could affect phosphorus handling at the intestine, kidney, and bone. In this study, only male rats were used; thus, we are unable to determine sex differences in intestinal phosphorus absorption efficiency nor generalize our results to females. Moreover, the translation of findings from rodent models to humans must further be elucidated.^(^ ^55^ ^)^ The major strength of this study was the use of the in vivo oral gavage technique to determine intestinal fractional phosphorus absorption efficiency, as this technique most accurately replicates physiological intestinal absorption compared with ex vivo or in vitro methods. Further, the incorporation of blinded randomization of rats to each group minimized the risk for bias.

In conclusion, the present study investigated how an acute high‐phosphorus load after a low‐phosphorus diet affected intestinal phosphorus absorption efficiency in 5/6 nephrectomized and sham‐operated male rats using an in vivo oral gavage technique. Acute high phosphorus did not lead to enhanced fractional phosphorus absorption efficiency, which provides evidence against the notion that dietary phosphorus restriction adversely increases absorption efficiency during dietary non‐adherence, at least short term. However, high‐phosphorus loads do result in greater absolute phosphorus absorption and plasma phosphorus levels. Our data support continued efforts to limit phosphorus intake in patients with CKD. However, clinical studies are needed to confirm these findings in all stages of CKD and including cardiovascular endpoints.

## Conflicts of Interest

KMHG has received speaker honoraria from Ardelyx and scientific consulting fees from Tricida. CJV has received honoraria from The Obesity Society and the Alliance for Potato Research and Education. The institution of CJV, Indiana University, has received funds to support his research from National Cattlemen's Beef Association; Alliance for Potato Research and Education; the Gordon and Betty Moore Foundation; and NIH. AB has received research grants from Keryx Pharmaceuticals and honoraria from Amgen. SMM is a scientific consultant for Sanifit, Amgen, and Ardelyx. KMB, DPC, JMD, NXC, KO, and MEW have nothing to disclose.

## Author Contributions

**Kendal M Burstad:** Data curation; formal analysis; investigation; visualization; writing – original draft; writing – review and editing. **Dennis P Cladis:** Data curation; investigation; writing – review and editing. **Colby J Vorland:** Conceptualization; investigation; methodology; visualization; writing – review and editing. **Meryl E Wastney:** Formal analysis; investigation; methodology; writing – review and editing. **Annabel Biruete:** Data curation; investigation; writing – review and editing. **James M Dominguez II:** Data curation; investigation; writing – review and editing. **Kalisha D O'Neill:** Data curation; investigation; writing – review and editing. **Neal X Chen:** Data curation; investigation; writing – review and editing. **Sharon M Moe:** Methodology; resources; supervision; writing – review and editing. **Kathleen M Hill Gallant:** Conceptualization; formal analysis; funding acquisition; investigation; methodology; project administration; resources; supervision; writing – review and editing.

## Supporting information


**Supplemental Table S1.** Study Diet Formula
**Supplemental Table S2.** Day 7 Food Consumption and Final Body Weight
**Supplemental Table S3.** Intestinal Fractional Phosphorus Absorption and Endpoint Plasma Biochemistries by Health Status and Diet Treatment
**Supplemental Table S4.** Intestinal Phosphate Transporters Gene Expression by Health Status and Diet Treatment
**Supplemental Figure S1.** Oral dose curves.
**Supplemental Figure S2.** IV dose curve.
