# Supplementary material for: Acute High Dietary Phosphorus Following Low‐Phosphorus Diet Acclimation Does Not Enhance Intestinal Fractional Phosphorus Absorption in Nephrectomized Male Rats
Source: JBMR Plus. 2022 Nov 16;6(12):e10698. doi: 10.1002/jbm4.10698 (PMC9751657; doi:10.1002/jbm4.10698)
Supplement: Supplementary file 1 — Supplemental Table S1. Study Diet Formula Supplemental Table S2. Day 7 Food Consumption and Final Body Weight Supplemental Table S3. Intestinal Fractional Phosphorus Absorption and Endpoint Plasma Biochemistries by Health Status and Diet Treatment Supplemental Table S4. Intestinal Phosphate Transporters Gene Expression by Health Status and Diet Treatment Supplemental Figure S1. Oral dose curves. Supplemental Figure S2. IV dose curve. [file JBM4-6-e10698-s001.docx]

**Supplementary Table 1: Study Diet Formula**

|  | TD.85010 (LP, 0.1% P)  (g/kg) | TD.85349 (HP, 1.2% P)  (g/kg) |
| --- | --- | --- |
| Egg White Solids | 200 | 200 |
| Sucrose | 525.2 | 501.2 |
| Corn Starch | 150 | 150 |
| Corn Oil | 50 | 50 |
| Cellulose | 20 | 20 |
| Mineral Mix, Ca-P Deficient (79055) | 13.4 | 13.4 |
| Calcium Carbonate | 14.7 | 5.03 |
| Potassium Bicarbonate | 8.1 | - |
| Sodium Chloride | 4.7 | - |
| Sodium Phosphate, Monobasic, Monohydrate | 1.7 | 12.9 |
| Potassium Phosphate, Monobasic | 1.7 | 12.7 |
| Calcium Phosphate, Monobasic, Monohydrate | - | 24.4 |
| Vitamin Mix, Teklad (40060) | 10 | 10 |
| Biotin | 0.004 | 0.004 |
| Yellow Food Color | 0.3 | 0.3 |
| Blue Food Color | 0.15 | - |
| Red Food Color | - | 0.15 |
|  |  |  |
| Protein (% kcal from) | 17.2 | 17.6 |
| Carbohydrate (% kcal from) | 70.8 | 70.1 |
| Fat (% kcal from) | 12 | 12.3 |
| Kcal/g | 3.8 | 3.7 |

**Supplementary Table 1:** Study diets were formulated to contain either low (0.1% P w/w, TD.85010, Envigo Teklad, Indianapolis, IN, USA) or high phosphorus (1.2% P w/w, TD.85349 Envigo Teklad, Indianapolis, IN, USA).

**Supplementary Table 2: Day 7 Food Consumption and Final Body Weight**

|  | **Health Status** | | **Diet** | | | **Health Status x Diet** | | | | | | **ANCOVA P-Values** | | | |
| --- | --- | --- | --- | --- | --- | --- | --- | --- | --- | --- | --- | --- | --- | --- | --- |
|  |  |  |  |  |  | **LP** | | **LPHP** | | **HP** | |  |  |  |  |
|  | **Sham** | **CKD** | **LP** | **LPHP** | **HP** | **Sham** | **CKD** | **Sham** | **CKD** | **Sham** | **CKD** | **Model** | **Health**  **Status** | **Diet** | **HxD** |
| **Day 7 Food Consumption (g/d)**^1^ | 17  (4.34**)** | 19  (4.32) | 17  (4.32) | 17  (4.32) | 20  (4.38) | 17  (4.31) | 17  (4.16) | 15  (4.31) | 19  (4.16) | 19  (4.5) | 20  (4.31) | 0.25 | 0.12 | 0.12 | 0.24 |
| **Final Body Weight**  **(g)**^1^ | 371  (27.3)* | 340  (27.2) | 347  (27.3) | 361  (27.3) | 358  (27.3) | 362  (27.2) | 333  (27) | 369  (27.2) | 353  (27) | 381  (27.3) | 335  (27.2) | **0.0005** | **<0.0001** | 0.21 | 0.18 |

**Supplementary Table 2:** ANCOVA p-values for the overall model (*P*_Model_), main of effect of health status (*P*_Health_), diet (*P*_Diet_), and their interaction (*P*_HxD_) for day 7 food consumption and final body weight. LS means ± SD are shown. Food consumption did not differ between groups. Final body weight was lower in CKD compared to sham rats, as expected due to CKD.

^1^n= 11 sham LP, 11 sham LPHP, 9 sham HP, 12 CKD LP, 12 CKD LPHP, 11 CKD HP

**Supplementary Figure 1. Oral Dose Curves**

**Supplementary Figure 1. A-F Oral Dose Curves:** Plasma ^33^P levels at each timepoint are shown as a percent of oral isotope dose for each individual rat. Each color per graph represents one rat. Values presented are from 100 µL of plasma counted at each time point over a 2-hour period. Axes for all graphs were kept consistent for ease of interpretation.

n= 7 sham LP, 7 sham LPHP, 6 sham HP, 7 CKD LP, 8 CKD LPHP, 8 CKD HP

**Supplementary Figure 2. I.V. Dose Curve**

**Supplementary Figure 2. A-F I.V. Dose Curves:** Plasma ^33^P levels at each timepoint are shown as a percent of I.V. isotope dose for each individual rat. Each color per graph represents one rat. Values presented are from 100 µL of plasma counted at each time point over a 2-hour period. Axes for all graphs were kept consistent for ease of interpretation.

n= 4 sham LP, 4 sham LPHP, 3 sham HP, 4 CKD LP, 4 CKD LPHP, 3 CKD HP

**Supplementary Table 3: Intestinal Fractional Phosphorus Absorption and Endpoint Plasma Biochemistries by Health Status and Diet Treatment**

|  | **Health Status** | | **Diet** | | | **ANCOVA P-Values** | | | |
| --- | --- | --- | --- | --- | --- | --- | --- | --- | --- |
|  | **Sham** | **CKD** | **LP** | **LPHP** | **HP** |  | | | |
|  |  |  |  |  |  | **Model** | **Health**  **Status** | **Diet** | **HxD** |
| **Intestinal Fractional Phosphorus Absorption (AUC_PO_/AUC_IV_)**^1^ | 0.09  (0.04) | 0.11  (0.05) | 0.14  (0.04) | 0.09  (0.04) | 0.09  (0.04) | 0.12 | 0.31 | **0.03** | 0.97 |
| **BUN (mg/dL)**^2^ | 22.5  (5.12) | 38.9  (5.09)* | 31.8  (5.13) | 30.6  (5.13) | 29.7  (5.14) | **<0.0001** | **<0.0001** | 0.40 | 0.73 |
| **P (mg/dL)**^3^ | 9.7  (1.17) | 10.4  (1.15) | 5.8  (1.15) | 12.5  (1.13) | 12.0  (1.13) | **<0.0001** | **0.02** | **<0.0001** | **<0.0001** |
| **Ca (mg/dL)**^4^ | 10.7  (1.15) | 10.6  (1.12) | 11.3  (1.15)^a^ | 9.8  (1.17)^b^ | 11.0  (1.16)^a^ | **<0.0001** | 0.71 | **0.0001** | 0.08 |
| **iFGF23 (pg/mL)**^5^ | 242  (93.1) | 403  (139) | 153  (51.3) | 380  (142.6) | 523  (177.8) | **<0.0001** | **<0.0001** | **<0.0001** | **0.006** |
| **iPTH (pg/dL)**^6^ | 1456  (642.5) | 1355  (633.6) | 707  (634)^c^ | 1337  (634)^b^ | 2174  (642.1)^a^ | **<0.0001** | 0.53 | **<0.0001** | 0.12 |
| **1,25(OH)_2_D_3_(pg/mL)**^7^ | 147  (50.3) | 145  (50.1) | 138  (50.5) | 149  (50.2) | 151  (50.5) | 0.53 | 0.87 | 0.68 | 0.51 |

**Supplementary Table 3:** LS means ± SD for intestinal fractional phosphorus absorption and plasma biochemistries by health status and diet treatment. *represents <0.0001 between health status. Different superscripted letters represent group differences.

^1^n= 7 sham LP, 7 sham LPHP, 6 sham HP, 7 CKD LP, 8 CKD LPHP, 8 CKD HP

^2^n= 11 sham LP, 11 sham LPHP, 9 sham HP, 12 CKD LP, 12 CKD LPHP, 11 CKD HP

^3^n= 11 sham LP, 11 sham LPHP, 9 sham HP, 12 CKD LP, 11 CKD LPHP, 10 CKD HP

^4^n= 11 sham LP, 10 sham LPHP, 9 sham HP, 12 CKD LP, 12 CKD LPHP, 11 CKD HP

^5^n= 11 sham LP, 10 sham LPHP, 9 sham HP, 12 CKD LP, 12 CKD LPHP, 9 CKD HP

^6^n= 11 sham LP, 11 sham LPHP, 8 sham HP, 12 CKD LP, 12 CKD LPHP, 11 CKD HP

^7^n= 9 sham LP, 10 sham LPHP, 9 sham HP, 11 CKD LP, 12 CKD LPHP, 11 CKD HP

**Supplementary Table 4: Intestinal Phosphate Transporters Gene Expression by Health Status and Diet Treatment**

|  | **Health Status** | | **Diet** | | | **ANCOVA P-Values** | | | |
| --- | --- | --- | --- | --- | --- | --- | --- | --- | --- |
|  | **Sham** | **CKD** | **LP** | **LPHP** | **HP** |  | | | |
|  |  |  |  |  |  | **Model** | **Health Status** | **Diet** | **HxD** |
| **Duodenal NaPi-2b/RPLP0**^1^ | 2.10  (2.12) | 1.96  (1.87) | 2.13  (1.68) | 1.80  (1.31) | 2.19  (1.43) | 0.27 | 0.72 | 0.71 | 0.17 |
| **Duodenal Pit-1/RPLP0**^2^ | 1.10  (0.61) | 1.04  (0.58) | 1.02  (0.58) | 1.04  (0.58) | 1.14  (0.65) | 0.49 | 0.66 | 0.82 | 0.0501 |
| **Duodenal Pit-2/RPLP0**^a1^ | 1.11  (0.39) | 1.12  (0.35) | 0.9  (0.38)^b^ | 1.37  (0.38)^a^ | 1.07  (0.36)^b^ | **0.01** | 0.91 | **0.0002** | 0.64 |
| **Jejunal NaPi-2b/RPLP0**^3^ | 2.61  (2.53) | 2.36  (2.33) | 2.92  (2.98)^a,b^ | 1.46  (1.39)^b^ | 3.60  (3.62)^a^ | **0.007** | 0.69 | **0.01** | 0.18 |
| **Jejunal Pit-1/RPLP0**^4^ | 2.36  (2.05) | 2.77  (2.48) | 2.80  (2.53) | 2.1  (1.92) | 2.89  (2.53) | 0.33 | 0.47 | 0.41 | 0.16 |
| **Jejunum Pit-2/RPLP0**^5^ | 1.60  (0.71) | 1.39  (0.59) | 1.46  (0.62) | 1.55  (0.67) | 1.46  (0.65) | **0.0003** | 0.19 | 0.86 | 0.98 |

**Supplementary Table 4:** LS means ± SD for intestinal phosphate transporters gene expression by health status and diet treatment. Different superscripted letters represent group differences.

^1^n = 11 sham LP, 11 sham LPHP, 9 sham HP, 12 CKD LP, 11 CKD LPHP, 11 CKD HP

^2^n= 11 sham LP, 11 sham LPHP, 9 sham HP, 12 CKD LP, 12 CKD LPHP, 10 CKD HP

^3^n= 10 sham LP, 11 sham LPHP, 8 sham HP, 11 CKD LP, 12 CKD LPHP, 11 CKD HP

^4^n= 10 sham LP, 10 sham LPHP, 8 sham HP, 12 CKD LP, 12 CKD LPHP, 11 CKD HP

^5^n= 11 sham LP, 10 sham LPHP, 8 sham HP, 12 CKD LP, 12 CKD LPHP, 11 CKD HP
